# The provision of chiropractic, physiotherapy and osteopathic services within the Australian private health-care system: a report of recent trends

**DOI:** 10.1186/2045-709X-22-3

**Published:** 2014-01-15

**Authors:** Roger M Engel, Benjamin T Brown, Michael S Swain, Reidar P Lystad

**Affiliations:** 1 Department of Chiropractic, Macquarie University, Balaclava Rd, North Ryde 2109, Australia

**Keywords:** Chiropractic, Physiotherapy, Osteopathy, Allied health, Healthcare utilisation

## Abstract

**Background:**

Chiropractors, physiotherapists, and osteopaths receive training in the diagnosis and management of musculoskeletal conditions. As a result there is considerable overlap in the types of conditions that are encountered clinically by these practitioners. In Australia, the majority of benefits paid for these services come from the private sector. The purpose of this article is to quantify and describe the development in service utilization and the cost of benefits paid to users of these healthcare services by private health insurers. An exploration of the factors that may have influenced the observed trends is also presented.

**Methods:**

A review of data from the Australian Bureau of Statistics, Australian Health Practitioner Regulation Agency, and the Australian Government Private Health Insurance Administration Council was conducted. An analysis of chiropractic, physiotherapy and osteopathic service utilisation and cost of service utilisation trend was performed along with the level of benefits and services over time.

**Results:**

In 2012, the number of physiotherapists working in the private sector was 2.9 times larger than that of chiropractic, and 7.8 times that of the osteopathic profession. The total number of services provided by chiropractors, physiotherapists, and osteopaths increased steadily over the past 15 years. For the majority of this period, chiropractors provided more services than the other two professions. The average number of services provided by chiropractors was approximately two and a half times that of physiotherapists and four and a half times that of osteopaths.

**Conclusions:**

This study highlights a clear disparity in the average number of services provided by chiropractors, physiotherapists, and osteopaths in the private sector in Australia over the last 15 years. Further research is required to explain these observed differences and to determine whether a similar trend exists in patients who do not have private health insurance cover.

## Introduction

In Australia health care costs are covered by the private and public sectors. In 2009–2010 the private health insurance sector provided $13.5 billion towards the total cost of the Australian health care system. This represented about 12% of total health funding [1]. These funds came from two sources: insurance premium payments by members ($9.2 billion) and premium rebates from the Australian Government ($4.3 billion). Of the $9.2 billion paid by members, 5.3% went to payment for non-hospital, non-medical treatment such as chiropractic, physiotherapy and osteopathic services.

Australian chiropractors, physiotherapists and osteopaths are all trained to diagnose and treat musculoskeletal (MSK) conditions [2-4]. It is therefore reasonable to assume a degree of overlap in the types of conditions treated by each of these professions. A recent cross-sectional study of chiropractors from Victoria, Australia reported that MSK conditions were the most common reason for patients seeking chiropractic care. Chiropractors who participated in the study stated that MSK conditions were the main reason for the practitioner-patient encounter in 60 per 100 encounters (95% CI, 54–67 per 100 encounters) [5]. The Australian Physiotherapy Association’s ‘Scope of Practice’ states that physiotherapy involves the prevention, diagnosis, and management of movement disorders that involve the MSK system [6]. In addition, physiotherapists also provide other services including, but not limited to, post-fracture, post-surgery and pre- and post-natal care. It is a similar scenario for osteopaths with nine of the twelve conditions listed under ‘Treatment’ by the Australian Osteopathic Association being MSK in nature [3].

Musculoskeletal conditions such as back and neck pain cause significant disability and loss of productivity and represent a considerable portion of healthcare expenditure in Australia. The Global Burden of Disease study 2010 ranks low back pain and neck pain first and third respectively in Australasia as the cause of years lived with disability [7]. The prevalence of these conditions increases up to the age of 60 [8] and the demand for health care services associated with managing them is likely to increase significantly over the next three decades. It is therefore important to quantify and describe current trends in service utilisation in order to assist future planning of healthcare service provision and expenditure.

In Australia, the provision of chiropractic, physiotherapy and osteopathic services is driven by a number of factors that can be classified as internal or external. Internal factors encompass graduate capabilities, code of conduct and self-regulated professional behaviour and practice, while external factors include government legislation, private health insurance policies, individual/community preference, population demographics, economic circumstances such as the level of household disposable income and the health status of the population [9]. These factors operate in a state of dynamic equilibrium, whereby change in one factor produces an alteration in one or more of the others. Understanding the dynamics of these changes is not always straightforward. The aims of this article are to A) quantify and describe developments in service utilisation and total cost of service benefits for chiropractic, physiotherapy and osteopathic services in the Australian private health sector, and B) to discuss factors that may account for any observable trends in the development.

## Methods

### Data sources

Data were obtained retrospectively from the Australian Bureau of Statistics (ABS) [10], Australian Health Practitioner Regulation Agency (AHPRA) [11-13], and the Australian Government Private Health Insurance Administration Council (PHIAC) [14,15] with a review period of 15 years (1998–2012) chosen for convenience. The PHIAC is the regulatory body for the private health insurance industry in Australia. It protects the consumer by ensuring a safe, efficient, compliant, and competitive health insurance industry. The PHIAC collects key information and monitors activity from all health insurers in Australia on a quarterly basis.

In this study data was gathered from PHIAC pertaining to total cost of services, ancillary benefits, and number of services provided to all Australians with private health insurance by chiropractors, physiotherapists and osteopaths. The total ‘cost of service’ represents the sum total of the fees charged at the time of consultation (fee for service) to individuals with private health insurance. An ‘ancillary benefit’ relates to the portion of the total fee for service that is reimbursed by the health insurer. A ‘service’ represents one visit to a healthcare provider. The number of general treatment policies held during the review period was also retrieved from the PHIAC databases. A ‘general treatment policy’ represents insurance for treatment that is intended to manage or prevent a disease, injury or condition, and is separate to hospital treatment cover.

Total numbers of registered, practising practitioners in 1998 and 2012 were collated using data from the ABS and AHPRA. Data regarding the total number of hours worked by registered physiotherapists, in both the public and the private sector, were obtained from the ABS and from three Australian state government agencies (New South Wales 2009, Queensland 2005, and Victoria 2006) [16-19]. The ABS data described the percentage of hours worked by physiotherapists in the private sector in 1998.

Quarterly data relating to the majority of these key variables was available for the review period for the chiropractic, physiotherapy and osteopathic professions. In this study, quarterly services, cost of services, and ancillary benefits data were summated to give yearly statistics for that period. No other transformations were required.

### Data analyses

Each discipline’s service utilisation and cost of service utilisation trend was plotted showing the level of services and benefits over time. The percentage change from one year to the next for both total service cost and number of services was calculated for chiropractic, physiotherapy and osteopathy. The percentage change in practitioner numbers between 1998 and 2012 was also calculated. As chiropractors and osteopaths work exclusively in the private sector, an estimate of the number physiotherapists working in the private sector was required. The estimate for the total number of physiotherapists working in the private sector in 2012 was based on data from the three state government agencies. The proportion of 53.5% was calculated by taking the average number of hours worked by physiotherapists in the private sector across these three states and applying it to the total number of registered, practising physiotherapists in 2012. The average yearly number of services delivered was calculated for each profession by dividing the total number of services by the total number of registered, practising practitioners in the private sector for each profession. The percentage change from the previous yearly figures was calculated for all the key variables.

## Results

### Number of practitioners

Table 1 shows the number of chiropractors, physiotherapists and osteopaths in Australia in 1998 and 2012 working in private practice and the proportional change over time [11-13,19-21]. As the number of physiotherapists working in the private sector in 2012 was not directly available we used the estimate referred to previously for the analysis. There were just under three times (2.9) as many physiotherapists as chiropractors in 2012 and 2.6 times as many chiropractors as osteopaths. The osteopathic profession experienced a four-fold increase in practitioner numbers during the review period which was considerably larger than the increases in the number of chiropractors and physiotherapists. It is however important to note that despite this large increase in the practitioner numbers during the review period, the overall number of osteopaths is much smaller when compared to the number of chiropractors and physiotherapists working in the private sector.

**Table 1:** Total number of registered, practising practitioners in the private sector by profession in 1998 and 2012

**Provider**	**1998**	**2012**	**Proportion change**
Chiropractors	2,053	4,221	2.1
Physiotherapists	5,187	12,230^*^	2.4
Osteopaths	395	1,608	4.1

*Estimated figure: 53.5% of total registered, practising physiotherapists (24,304).

**Source:** 1998 figures ABS [19,21], 2012 figures AHPRA [11-13].

### Level of services

**Figure 1:**
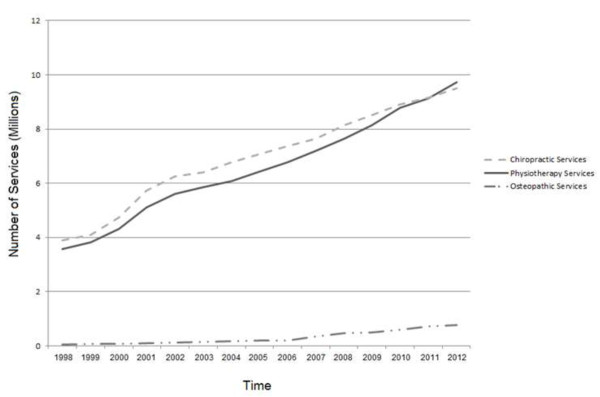
**Total number of services provided to individuals with private health insurance by profession: 1998 – 2012.** Source: PHIAC [15].

**Figure 2:**
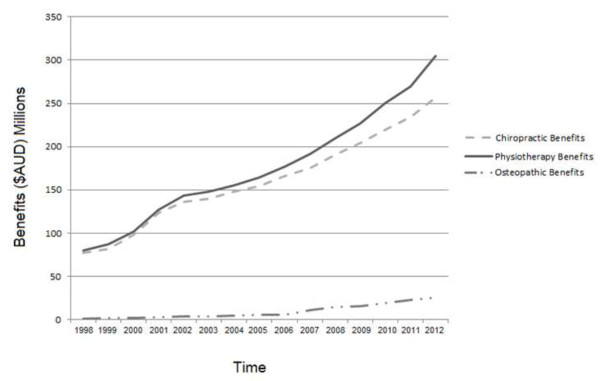
**Private health insurance rebates by profession: 1998–2012.** Source: PHIAC [15].

Figure 1 reports the total number of chiropractic, physiotherapy and osteopathic services provided to individuals with private health insurance between 1998 and 2012 [22]. Profession service figures have been provided in Additional file 1[15]. Figure 2 illustrates the total private health insurance expenditure for chiropractic, physiotherapy and osteopathic services between 1998 and 2012. A detailed breakdown of this data is provided in Additional file 2[22]. There was an overall trend towards increasing expenditure for all three providers during the review period. While the total number and dollar value of osteopathic services was less compared to chiropractic and physiotherapy services the percentage change in osteopathic services exceeded chiropractic and physiotherapy for the majority of the review period. The expenditure on osteopathic services must be interpreted with the knowledge that the overall level of services for the osteopathic profession represents a small percentage of the total expenditure for services rendered by the three services providers.

### Average number of services per practitioner

In 1998 the average number of services per practitioner provided to individuals with private health insurance per practitioner was 1,901 for chiropractors, 691 for physiotherapists and 141 for osteopaths. Using the estimated figure for the number of physiotherapists working in the private sector, the average number of services per registered, practising practitioner in 2012 was 2,252 for chiropractors, 796 for physiotherapists and 483 for osteopaths [11-13]. The average number of services provided by chiropractors was more than two and a half times that of physiotherapists and four and a half times that of osteopaths working in the private sector in 1998 and 2012.

## Discussion

The purpose of this study was to quantify and describe developments in service utilisation and total cost of service benefits for chiropractic, physiotherapy and osteopathic services in the private healthcare sector in Australia between 1998 and 2012. There has been a substantial increase in the number of chiropractors, physiotherapists and osteopaths in the last 15 years, with physiotherapy being the largest of the three providers in terms of practitioner numbers in the private sector. This growth in practitioner numbers has been accompanied by a steady increase in the utilisation of these services, a scenario that has been most pronounced for osteopathic services. Chiropractors have delivered more services to individuals with private health insurance compared to physiotherapists and osteopaths for the majority of the review period. Chiropractors deliver, on average, a higher number of services each year in the private sector compared to physiotherapists and osteopaths. With respect to expenditure, there has been a trend towards an increasing cost of chiropractic, physiotherapy and osteopathic services, as well as an increase in the benefits paid for these services by private health insurers. Both the total cost and the benefits paid for osteopathic services have accelerated dramatically over the review period when viewed alongside the data for the other two service providers.

The secondary aim of this study was to discuss the factors that may account for the development in the figures relating to total cost of services, benefits paid by private health insurers, and utilisation rates for chiropractic, physiotherapy and osteopathic services. While it is not possible, based on the data, to provide definitive explanations for the observed trends a discussion of the factors that may have contributed to changes in these areas is presented below.

With respect to service utilisation, the increases observed during the review period cannot be fully explained by the increase in practitioner numbers. Between 2005 and 2010, the total number of workers across all health occupations in Australia increased by 26%. During the same period, the number of chiropractors and osteopaths increased by 17% while the number of physiotherapists increased by 42% [23].

The number of chiropractic services provided to patients with private health insurance was higher than for physiotherapy for the majority of the review period (1998–2011). If the two professions were compared on size alone it would make sense that the profession with the largest private sector presence (physiotherapy) would have also provided the most number of services. However, this was not the case for the majority of the review period. Data from 2012 highlights a change to this trend with the total number of services provided by physiotherapists exceeding those provided by chiropractors. It is yet to be seen whether this increase will continue into the future.

Factors that could explain the disparity in the number of services provided by chiropractors and physiotherapists during the review period include internal factors such as code of conduct and self-regulated professional behaviour and practice, and/or external factors such as government legislation, changes in private health insurance policy or economic factors.

Code of conduct and self-regulated professional behaviour and practice includes elements such as fees charged for services, and the location of practices. With respect to the fees charged for chiropractic services, the average fee declined in real terms between 2007 and 2012 [9]. The opposite situation occurred regarding fees for physiotherapy services with average fee in real terms increasing between 2007 and 2012 [24]. However, the benefit paid by private health insurers declined by approximately 1% in the same period for both chiropractic and physiotherapy services [9,24]. The size of the difference in both fee for service, and benefits paid by insurers over this period is therefore unlikely to account for the disparity between the average number of chiropractic and physiotherapy services.

While there is a general consensus regarding the types of conditions that fall within the scope of practice for each profession, the precise case-mix for each profession is not well-described in the literature. It could be argued that physiotherapists, through their involvement in areas such as post-surgical rehabilitation, may see patients who justifiably require more frequent and intensive care. This could have created a scenario where there is considerable heterogeneity in case-mix between the three professions. This scenario could also potentially alter the length of time required to treat each patient, with patients seeing a physiotherapist experiencing greater disability requiring more care and attention equating to longer ‘service’ duration.

The data presented in this study describe a higher average number of services provided per chiropractic practitioner compared to physiotherapists and osteopaths. This finding does not necessarily mean that chiropractors delivered more services per patient. Instead, it may be that chiropractors see more new patients or a greater proportion of their existing client base more often. Recent industry estimates suggest that Australian chiropractors see approximately 300,000 patients per week [9]. Using this estimate, each chiropractor (4402 in June 2013) would be seeing on average 68.1 patients per week. Based on a 48 week working year these estimates are higher than the estimates detailed in this study. More accurate point prevalence data on service utilization for each of these professions is required before any meaningful predictions can be made about the level of chiropractic, physiotherapy and osteopathic services in the future.

Results from a 2005 Australian survey of 1,067 adults over the age of 18 found that 16.1% reported using chiropractic services during the previous 12 months [25]. The most common reasons given for seeking chiropractic and osteopathic treatment were back and/or neck pain, non-specific musculoskeletal problems and enhancement of general health and well-being [25]. While the response rate for each of these categories did differ between the two professions the size of the differences are not enough to explain the disparity in the average number of services for each profession.

A recent survey of 1,427 Australian women showed high utilisation of chiropractors in non-urban compared to urban settings [26]. It may be the case that there is a different pattern in the utilisation of chiropractic, physiotherapy and osteopathic services in rural areas. It is important to note that there has not been any research into the utilisation of these services by males in non-urban regions. However, we do not believe that a preference for chiropractic in non-urban settings would be sufficient to explain the magnitude of the disparity in average servicing as the number of chiropractors practising in rural and remote regions of Australia (30%) is substantially less than the number of chiropractors practising in urban regions (70%) [27].

With respect to the previously identified external factors that could explain the utilisation disparity between the three disciplines, the Australian Federal Government does not regulate the level of chiropractic, physiotherapy or osteopathic services provided in the private sector. As for private health insurance coverage, the increased uptake in general treatment cover over the review period does not match the increase in service provision by the three professions. There have not been any changes in private health insurance policies during the review period that favoured one discipline over the other or any economic factors that may have influenced the Australian economy in a way that would have impacted on the three disciplines differently.

An absence of practice guidelines for the chiropractic profession could account for a high average number of services provided per practitioner. This absence may have unwittingly led to the acceptance of a wide variation in the level of servicing among chiropractors in Australia. However, the problem is not unique to the chiropractic profession, and would therefore exist to some extent in both the physiotherapy and osteopathic professions. This is an area in which further research is required. While our analysis cannot identify whether the average number of services per chiropractor can be uniformly applied across the profession, it is possible that a section of the profession practices in a manner so different to the rest that the average for the entire profession becomes distorted. Anecdotally, there are large differences between chiropractors in the average number of services provided per patient. It must be acknowledged that the same scenario could also exist within the physiotherapy and osteopathic professions. However, if this were the case for chiropractic, it would be of interest to the private health insurance sector as they provide the majority of benefits for chiropractic services. In the current economic climate, it is hard to imagine insurers continuing to support such high levels of utilisation without evidence to support the premise that a higher level of servicing delivers increased benefit to their members. This point is especially pertinent to insurance companies as there has been a steady increase in the number of Australians with general treatment insurance over the review period [15]. It must be noted however that while an individual may have had general treatment cover, this does not mean that they have coverage for chiropractic, physiotherapy, or osteopathic treatment within that general treatment cover.

The differences observed between the three service providers with respect to service utilisation, and average number of services provided per practitioner cannot be adequately explained by the data presented here. It is possible that factors that were not measured in this research such as working hours, full-time equivalent working status, capacity of a profession to treat patients, patient demographics, and treatment type/content may be salient. Further research that includes these metrics is required to more accurately account for the observed trends.

### Limitations

There are several limitations to this study. Firstly, the estimate for the number of physiotherapists working in the private sector was based on figures from only three states at different time periods. It is unknown how these estimates relate to full-time practitioners as there are no current reports that detail the full-time equivalent status of practitioners working in these three professions. Secondly, PHIAC collects data on the cost of benefits for services and the number of services received by individuals with private health insurance. However, if a patient had exceeded their yearly benefit limit for a particular service, any additional services provided by a treating practitioner after that point would not have been recorded. Furthermore, not all Australians have private health insurance. In fact, only 54.6% of Australians had general treatment policies in December 2012 [28]. Of the patients with private health insurance, not all had a level of cover that included ancillary services such chiropractic, physiotherapy or osteopathy. Currently, chiropractic, physiotherapy, and osteopathy services are bundled together in the majority of ancillary health cover packages. Patients are therefore not given the option to select health insurance coverage for an individual profession, despite any preference they may have. The reasons for seeking chiropractic treatment are quite diverse and readers are directed to a recent review paper by Hurwitz [29] for a more detailed discussion on the topic. It must be acknowledged that the data presented in this paper does not fully reflect the various reasons for why patients choose a particular modality and that this may be a salient factor in the observed trends in service utilization. Further research, including an analysis of the social and cultural reasons that drive care seeking behaviour, is required to accurately profile/explain the trends in utilisation across the three professions.

The data presented in this study do not include data from the Chronic Disease Management arm of the Enhanced Primary Care Program. Under this program, patients with a chronic medical condition, e.g. chronic musculoskeletal condition, who would benefit from multidisciplinary care, are eligible to receive five allied health services in conjunction with standard GP care. These services are rendered under a GP management plan and are covered publicly under the Medical Benefits Schedule in Australia [30]. Services provided under this program are not included in the data from PHIAC. It is possible that a proportion of patients that would ordinarily be recorded in the private system have not been identified in this study due to their involvement in this program. This may have influenced the figures relating to total cost of services, level of services and the number of average services provided per practitioner. It is however important to note that chiropractors, physiotherapists and osteopaths are involved in this program.

The estimate for the point prevalence of chiropractic service utilisation obtained in this study is lower than the utilisation figures obtained from a recent industry report [9]. This finding highlights a need for accurate data regarding point prevalence of chiropractic service utilisation. There is also a paucity of data pertaining to point prevalence of service utilisation for physiotherapy and osteopathy services. Targeted research into service utilisation rates amongst those with and without private health insurance is required to accurately explain the observed trends in service utilisation for these three professions.

## Conclusion

This article describes the development in the utilisation of chiropractic, physiotherapy and osteopathic services in Australia over the past 15 years. While there is a paucity of data to inform the drivers of this change the authors have provided some hypotheses for the identified disparity in service provision and identified areas where further research is warranted. While this study highlights a clear disparity in service utilisation in the Australian private sector, further research is required to determine if a similar trend exists in patients who do not have private health insurance cover. De-identified individual patient data and de-identified individual clinician data regarding variability in frequency of services per patient are required to better explain the observed trends.

## Abbreviations

ABS: Australian Bureau of Statistics; AHPRA: Australian Health Practitioner Regulation Agency; PHIAC: Private Health Insurance Administration Council; MSK: Musculoskeletal.

## Competing interests

The authors declare that they have no competing interests.

## Authors’ contributions

All authors contributed equally to the design, analysis, writing, and review of the final manuscript.

## Supplementary Material

Additional file 1 Number of services provided by profession: 1998 - 2012. Click here for file

Additional file 2 Total cost ($AUD) of services provided by profession: 1998 - 2012. Click here for file
