# Supplementary material for: The provision of chiropractic, physiotherapy and osteopathic services within the Australian private health-care system: a report of recent trends
Source: Chiropr Man Therap. 2014 Jan 15;22:3. doi: 10.1186/2045-709X-22-3 (PMC3896731; doi:10.1186/2045-709X-22-3)
Supplement: Additional file 1 — Number of services provided by profession: 1998 - 2012. [file 2045-709X-22-3-S1.docx]

**Additional File 1:**

**Number of services provided by profession: 1998 - 2012**

Figures derived from the private health insurance industry and represent the number of services provided to individuals with private health insurance. The percentage change from the previous year is also presented. Services provided to individuals who do not have private health insurance are not represented here.

| **Year** | **Chiropractic** | | **Physiotherapy** | | **Osteopathy** | |
| --- | --- | --- | --- | --- | --- | --- |
|  | **Services**  **(n)** | **Change**  **(%)** | **Services**  **(n)** | **Change**  **(%)** | **Services**  **(n)** | **Change**  **(%)** |
| **1998** | 3,903,511 | - 1.2 | 3,584,629 | - 2.1 | 55,821 | - 4.2 |
| **1999** | 4,090,320 | 4.8 | 3,821,879 | 6.6 | 69,826 | 25.1 |
| **2000** | 4,733,948 | 15.7 | 4,318,284 | 13.0 | 87,402 | 25.2 |
| **2001** | 5,737,333 | 21.2 | 5,123,617 | 18.6 | 108,436 | 24.1 |
| **2002** | 6,258,976 | 9.1 | 5,604,264 | 9.4 | 127,657 | 17.7 |
| **2003** | 6,407,940 | 2.4 | 5,855,110 | 4.5 | 151,841 | 18.9 |
| **2004** | 6,773,938 | 5.7 | 6,071,143 | 3.7 | 171,658 | 13.1 |
| **2005** | 7,076,987 | 4.5 | 6,429,509 | 5.9 | 192,197 | 12.0 |
| **2006** | 7,377,723 | 4.2 | 6,776,951 | 5.4 | 211,643 | 10.1 |
| **2007** | 7,630,833 | 3.4 | 7,191,787 | 6.1 | 356,554 | 68.5 |
| **2008** | 8,141,797 | 6.7 | 7,648,683 | 6.4 | 468,671 | 31.4 |
| **2009** | 8,498,796 | 4.4 | 8,138,529 | 6.4 | 491,943 | 5.0 |
| **2010** | 8,905,354 | 4.8 | 8,786,257 | 8.0 | 593,385 | 20.6 |
| **2011** | 9,162,619 | 2.9 | 9,129,596 | 3.9 | 712,209 | 20.0 |
| **2012** | 9,507,048 | 3.8 | 9,737,286 | 6.7 | 777,097 | 9.1 |

**Source:** PHIAC ^15^
