# Supplementary material for: The provision of chiropractic, physiotherapy and osteopathic services within the Australian private health-care system: a report of recent trends
Source: Chiropr Man Therap. 2014 Jan 15;22:3. doi: 10.1186/2045-709X-22-3 (PMC3896731; doi:10.1186/2045-709X-22-3)
Supplement: Additional file 2 — Total cost ($AUD) of services provided by profession: 1998 - 2012. [file 2045-709X-22-3-S2.docx]

**Additional File 2:**

**Total cost ($AUD) of services provided by profession: 1998 - 2012**

These figures are from the private health insurance industry and represent the total cost of services provided to individuals with private health insurance. The percentage change in the total cost of services relative to the previous year is also presented. Total cost represents the total fee for service for all services provided to individuals with private health insurance. Rebates to the patient from the private health insurer represent a percentage of the total fee for service and are not presented here. The total cost of services provided to individuals who do not have private health insurance are not presented here.

| **Year** | **Chiropractic** | | **Physiotherapy** | | **Osteopathy** | |
| --- | --- | --- | --- | --- | --- | --- |
|  | **Cost**  **($AUD)** | **Change**  **(%)** | **Cost**  **($AUD)** | **Change**  **(%)** | **Cost**  **($AUD)** | **Change**  **(%)** |
| **1998** | 126,089,155 | 1.0 | 131,272,123 | 0.0 | 2,559,210 | -0.5 |
| **1999** | 135,035,206 | 7.1 | 144,203,018 | 9.9 | 3,302,703 | 29.1 |
| **2000** | 163,086,036 | 20.8 | 169,337,704 | 17.4 | 4,359,173 | 32.0 |
| **2001** | 205,060,368 | 25.7 | 208,350,227 | 23.0 | 5,780,028 | 32.6 |
| **2002** | 232,250,752 | 13.3 | 235,621,081 | 13.1 | 6,897,245 | 19.3 |
| **2003** | 244,529,429 | 5.3 | 249,294,175 | 5.8 | 8,570,130 | 24.3 |
| **2004** | 267,878,768 | 9.5 | 271,734,128 | 9.0 | 9,908,337 | 15.6 |
| **2005** | 288,314,702 | 7.6 | 297,298,173 | 9.4 | 11,472,524 | 15.8 |
| **2006** | 311,678,934 | 8.1 | 327,644,149 | 10.2 | 13,105,238 | 14.2 |
| **2007** | 334,704,726 | 7.4 | 364,483,958 | 11.2 | 22,949,342 | 75.1 |
| **2008** | 370,200,994 | 10.6 | 406,478,331 | 11.5 | 31,583,074 | 37.6 |
| **2009** | 399,748,587 | 8.0 | 448,858,604 | 10.4 | 34,083,666 | 7.9 |
| **2010** | 426,717,590 | 6.7 | 498,823,156 | 11.1 | 42,183,021 | 23.8 |
| **2011** | 478,146,207 | 12.1 | 540,966,071 | 8.4 | 51,895,494 | 23.0 |
| **2012** | 492,866,458 | 3.1 | 601,399,440 | 11.2 | 58,360,884 | 12.5 |

**Source:** PHIAC ^15^
